# Application of Engineered Zinc Finger Proteins Immobilized on Paramagnetic Beads for Multiplexed Detection of Pathogenic DNA

**DOI:** 10.4014/jmb.2106.06057

**Published:** 2021-07-09

**Authors:** Jiyoung Shim, Langley Williams, Dohyun Kim, Kisung Ko, Moon-Soo Kim

**Affiliations:** 1 Department of Chemistry, Western Kentucky University, Bowling Green, KY 42101, USA; 2 Department of Mechanical Engineering, Myongji University, Yongin 17058, Republic of Korea; 3 Department of Medicine, College of Medicine, Chung-Ang University, Seoul 06974, Republic of Korea

**Keywords:** Multiplexed double-stranded DNA detection, zinc finger proteins, magnetic beads, foodborne pathogen

## Abstract

Micro-scale magnetic beads are widely used for isolation of proteins, DNA, and cells, leading to the development of in vitro diagnostics. Efficient isolation of target biomolecules is one of the keys to developing a simple and rapid point-of-care diagnostic. A zinc finger protein (ZFP) is a double-stranded (ds) DNA-binding domain, providing a useful scaffold for direct reading of the sequence information. Here, we utilized two engineered ZFPs (Stx2-268 and SEB-435) to detect the Shiga toxin (stx2) gene and the staphylococcal enterotoxin B (seb) gene present in foodborne pathogens, *Escherichia coli* O157 and *Staphylococcus aureus*, respectively. Engineered ZFPs are immobilized on a paramagnetic bead as a detection platform to efficiently isolate the target dsDNA-ZFP bound complex. The small paramagnetic beads provide a high surface area to volume ratio, allowing more ZFPs to be immobilized on the beads, which leads to increased target DNA detection. The fluorescence signal was measured upon ZFP binding to fluorophore-labeled target dsDNA. In this study, our system provided a detection limit of ≤ 60 fmol and demonstrated high specificity with multiplexing capability, suggesting a potential for development into a simple and reliable diagnostic for detecting multiple pathogens without target amplification.

## Introduction

Facile and rapid analytical technology has been developed for diverse applications such as clinical diagnostics [1] , food analysis, and environmental monitoring over the past two decades [2]. The development of simple and robust technology for pathogen detection is still in great demand to manage outbreaks and reduce health risks associated with specific pathogen infection. The conventional identification method for detection of pathogens is time-consuming culture of bacteria [3-5]. Thus, molecular diagnostic technology is desirable for its rapid detection. In particular, polymerase chain reaction (PCR) technology has been established as a quick and sensitive technique [6]. Additionally, the combination of PCR with a DNA microarray has been studied for the multiplexed detection of pathogens and biomarkers [7-11]. However, DNA hybridization is costly and time-consuming, requiring complicated double-stranded (ds) DNA denaturation and subsequent renaturation with carefully designed primers or probes [7, 12, 13].

The most common Cys2-His2 zinc finger domain is a useful scaffold for the construction of customized DNA-binding proteins that can read the sequence information directly from dsDNA [14, 15]. Each zinc finger protein (ZFP) forms a ββα structure, in which two cysteine and histidine residues coordinate a zinc ion. Amino acid residues at positions -1, 3, and 6 on the a-helix recognition module interact with specific DNA bases [16]. Twenty-five of the thirty amino acids in the repeat fold around a zinc ion to form a finger and the remaining five amino acids (TGEK(R)P) supply a short consensus linker between consecutive fingers [17]. Each ZF domain recognizes three to four nucleotides of DNA [18-21] and ZF domains can be linked to form multi-finger proteins using a modular assembly approach [22]. This modular assembly approach enables the rapid construction of multi-finger domains for binding any desired DNA sequence with high specificity [22]. Previously, we have demonstrated direct detection of specific dsDNA using engineered ZFPs by employing SEER-LAC (Sequence-Enabled Reassembly of TEM 1 β-lactamase) system [23, 24] in a colorimetric method, and chemiluminescent assay [25]. Other studies have been conducted utilizing ZFPs to detect miRNA-21 [26] and DNA methylation [27].

Magnetic beads can be functionalized with target moieties for efficient separation and detection of target molecules in a fast and simple procedure [28]. Micro-scale magnetic beads can be easily detected using fluorescence microscopy, which is useful for quick and specific detection of various biomolecules such as cancer biomarkers and cells [1, 29]. Shim *et al*. [30] conjugated antibodies to magnetic nanoparticles for rapid and facile detection of *Salmonella* Typhimurium. Hayes *et al*. [31] demonstrated a fast interaction time when using magnetic particles for a heterogeneous immunoassay. Owing to a larger surface to volume ratio, this study utilized magnetic beads for immobilizing the engineered ZFPs, thus enabling more target DNA binding. Here, we have developed a rapid, direct, and multiplexed dsDNA detection method by magnetically isolating the bead-bound complex of immobilized ZFPs (Stx2-268 and SEB-435) and fluorophore-labeled pathogenic dsDNA. Foodborne pathogens, *E. coli* O157 and *Staphylococcus aureus*, were selected for developing this detection method. The detection of pathogenic dsDNA with ZFPs immobilized on magnetic beads has demonstrated high specificity along with multiplexing capability, suggesting a potential for development into a simple and direct point-of-care (POC) testing for pathogenic detection.

## Materials and Methods

### Construction, Expression, and Purification of ZFPs

All ZFPs were constructed, expressed, and purified as described in the previous study [23, 25]. Each ZFP was constructed by the modular assembly method using the Barbas set of modules [22]. The vector enables bacterial expression of the proteins as fusions with an N-terminal maltose binding protein (MBP) as a purification tag. Proteins were expressed in *E. coli* BL21 (Invitrogen) upon induction with 1 mM isopropyl β-D-1-thiogalactopyranoside (IPTG) at an OD_600_ of 0.6–0.8 for 3 h at 37°C. Cells were pelleted and resuspended in Zinc Buffer A (ZBA: 100 mM Tris base, 90 mM KCl, 1 mM MgCl_2_ and 100 mM ZnCl_2_ at pH 7.5) including 5 mM dithiothreitol (DTT) and 50 mg/ml RNase A. After sonication, proteins in cell lysate were applied to an amylose resin column pre-equilibrated with ZBA containing 5 mM DTT, washed with ZBA containing 2 M NaCl and ZBA containing 1 mM tris(2-carboxyethyl) phosphine (TCEP), and eluted in ZBA containing 10 mM maltose and 1 mM TCEP. Concentration and purity were assessed by Coomassie-stained polyacrylamide gel electrophoresis with sodium dodecyl sulfate (SDS-PAGE) using bovine serum albumin (NEB) standards. A purified protein was stored in ZBA containing 1 mM TCEP at 4°C until use.

### ZFP Conjugation on Magnetic Beads

The protein storage buffer was exchanged to ZHEPES buffer (pH 7.5) containing 20 mM 4-(2-hydroxyethyl)-1-piperazineethanesulfonic acid (HEPES), 0.1 mM ZnCl_2_, 30 mM KCl, 1 mM MgCl_2_, and 5 mM DTT at pH 7.5 using 10-kDa Amicon filter unit (MilliporeSigma, Burlington, MA) by centrifugation at 4,000 × *g* for 1 h at 4°C. The ZFP concentration was re-evaluated using a Nanodrop (ThermoFisher, Waltham, MA). The concentrated ZFP was conjugated on Dynabeads M-280 Tosylactivated (ThermoFisher, USA) according to the manufacturer's protocol. The magnetic beads are polystyrene beads coated with a polyurethane layer. 1 mg of the magnetic beads was washed with 35 ml buffer A (0.1 M borate buffer pH 9.5) twice and then incubated 12 -16 h with a mixture of 20 mg of ZFP, 30 ml of buffer A, and 20 ml of buffer C (3 M ammonium sulfate in Buffer B containing 0.1 M sodium phosphate buffer with 0.1 mM ZnCl_2_, pH 7.4) at 37°C with shaking at 250 rpm. After overnight incubation, the beads were incubated with buffer D (PBS pH 7.4 with 0.5% (w/v) BSA, 5 mM DTT, and 0.1 mM ZnCl_2_) for 1 h at 37°C with shaking at 250 rpm and washed thoroughly using buffer E (PBS at pH 7.4 with 0.1% (w/v) BSA, 5 mM DTT, and 0.1 mM ZnCl_2_) twice. Finally, 48 ml of buffer E was added and stored at 4°C until use.

### Multiple Target DNA Detection by ZFPs

**Fig. 1:**
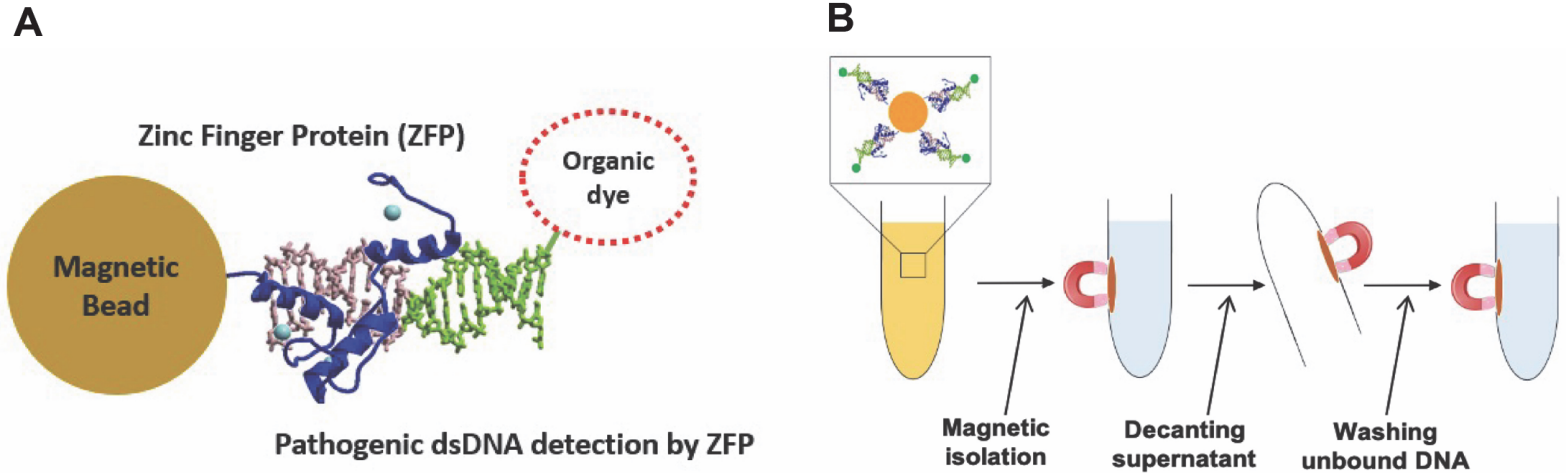
Schematic diagram of (**A**) the bead-bound complex of ZFP and fluorophore-labeled dsDNA on the magnetic beads and (**B**) magnetic isolation of the bead-bound complex before fluorescence measurement.

Single-stranded target DNA oligonucleotides were purchased from Integrated DNA Technologies (IDT, USA). Their sequences are provided in Table 1. We purchased dye-labeled target DNA oligonucleotides for ZFP Stx2-268 with Alexa 488 (excitation at 490 nm, emission at 520 nm) and ZFP SEB-435 with Texas red (excitation at 596 nm, emission at 620 nm), respectively. Target dsDNA oligonucleotides were prepared by heating at 95°C for 10 min and then slowly cooling down to ambient temperature to form hairpins containing a four-thymidine loop as described in the previous study [23, 25]. 5 μl of target dsDNA was applied to the ZFP-conjugated beads for binding. After a 20 min incubation at ambient temperature with gentle shaking, unbound DNA was washed away twice by magnetically isolating the bead-bound complex as illustrated in Fig. 1. The first wash buffer contains 50 mM KCl in ZBA buffer, and the second wash buffer contains 0.05% Tween-20 in ZBA buffer. The fluorescence intensity of the bead-bound complex was measured using a fluorescence microscope (Axiopan 2ie, Carl Zeiss, Germany). The intensity of a single bead on the fluorescence image was quantified using the NIH Image J software and data was statistically analyzed with the *t*-test.

## Results and Discussion

In this study, we employed paramagnetic beads with two ZFP probes, Stx2-268 and SEB-435, to develop a simple and rapid method for detection of pathogens. Magnetic beads could be isolated rapidly and provide a larger platform area to volume ratio, allowing for more ZFP molecule binding compared to the 2-dimentional surface. The magnetic beads used in this study are superparamagnetic and their size is uniformly 2.8 μm in diameter. The magnetic beads bind to ZFPs covalently through primary amino or sulfhydryl groups. After ZFPs conjugated on the beads bind target DNA form the protein-DNA bound complex, unbound molecules are washed out by magnetically isolating the bead-bound complex as illustrated in Fig. 1.

### Target DNA Detection on a Magnetic Bead-Based Platform

**Fig. 2:**
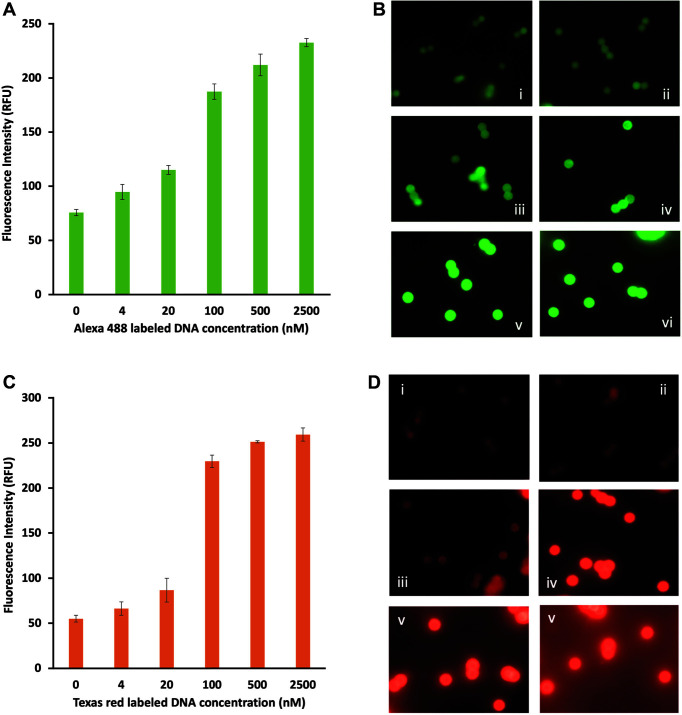
Target DNA detection with engineered ZFPs on a magnetic bead-based platform. (**A, C**) The bar graph representing the fluorescence intensity of individual particles obtained from (**B, D**) images taken by fluorescence microscopy (i: 0 nM, ii: 4 nM, iii: 20 nM, iv: 100 nM, v: 500 nM, and vi: 2500 nM DNA). (**A, B**) Alexa 488-labeled target DNA was detected by ZFP Stx2-268. (**C, D**) Texas red-labeled target DNA was detected by ZFP SEB-435.

Engineered ZFPs were highly expressed in *E. coli* and purified to approximately 90% purity. Engineered ZFP Stx2-268 binds to 18 bp of DNA sequence in the *stx2* gene encoding for the shiga toxin produced by *E. coli* O157. The ZFP SEB-435 engineered to recognize the *seb* gene encoding for the staphylococcal enterotoxin B in *S. aureus* was examined along with ZFP Stx2-268 in this study. To validate the magnetic bead-based detection platform, the ZFP assay was performed at various concentrations of target DNA oligonucleotides, ranging from 4 nM to 2.5 μM. The dsDNA of each target was applied to ZFPs immobilized on the magnetic beads. As the DNA concentration increases, the fluorescence signal obtained from individual beads using a fluorescence microscope increases, as shown in Fig. 2. Green fluorescent Alexa 488-labeled target DNA from the *stx2* gene was detected by ZFP Stx2-268 as shown in Fig. 2 (A, B). The fluorescence intensity of individual particles was obtained from the average of three independent experiments where data were collected ranging from 70 to 200 particles (1^st^ run), 60 to 200 particles (2^nd^ run), and 60 to 300 particles (3^rd^ run). The limit of detection was determined to be 4 nM for ZFP Stx2-268 (*p* < 0.05) which is equivalent to 60 fmol. Texas red-labeled target DNA from the *seb* gene was detected by ZFP SEB-435 as shown in Fig. 2 (C, D). The fluorescence intensity of individual particles was obtained from the average of three independent experiments where data were collected ranging from 150 to 200 particles (1^st^ run), 100 to 200 particles (2^nd^ run), and 100 to 300 particles (3^rd^ run). The result of the statistical analysis indicates that the ZFP SEB-435 was not significantly sensitive enough to detect ≤ 4 nM of target DNA. We found that the limit of detection was affected by the autofluorescence originating from the magnetic bead itself [32] as shown in the image i (No DNA) of Fig. 2 (B, D). The autofluorescence of the beads can increase background signal and reduces the assay sensitivity [32].

A potential improvement to this system could be the use of magnetic beads with minimal autofluorescence such as silicone magnetic beads [32], which could lead to enhanced sensitivity of the fluorescence-based assay. Also, further optimization of our system could improve the limit of detection by decreasing the amount of magnetic beads and increasing the amount of ZFP molecules. Although this magnetic bead-based platform is currently not as sensitive as the established leading nucleic acid-based methods such as PCR, our bead and ZFP-based detection system does not require additional steps involved in DNA denaturation and subsequent hybridization with carefully designed primers or probes due to the direct detection of dsDNA with customized ZFPs. In addition, our system does not require careful control of temperature nor expensive reagents needed for PCR. It should be noted that our system provides clear visual detection within a short assay time compared to an assay time of 2-3 h required for PCR. Our detection system lies in the utilization of flow based micro-scale assay along with a paramagnetic solid phase for immobilization and a magnet, which allows localized and convenient detection, small waste generation and reagent consumption, and a short overall assay time. Therefore, our system can be integrated into a microfluidic chip for developing a POC device. The beads coupled with a ZFP probe would be flown into a microfluidic chip and packed by a magnet placed underneath the chip. The fluorophore-labeled target DNA would then be pumped into the chip, thereby analyzing fluorescence signal of the protein-DNA bound complex. This application of ZFP array into a microfluidic module could lead to improvement in the sensitivity through pre-concentration of the sample.

The DNA binding affinities (k_D_) of ZFP Stx2-268 and ZFP SEB-435 are 1.98 and 0.3 nM, respectively as shown in Table 2 [23, 25]. While ZFPs with a stronger binding affinity may retain target DNA for a longer time, our observation may not allow for a clear correlation between the binding affinity of ZFPs and their detection characteristics.

### Specificity

**Fig. 3:**
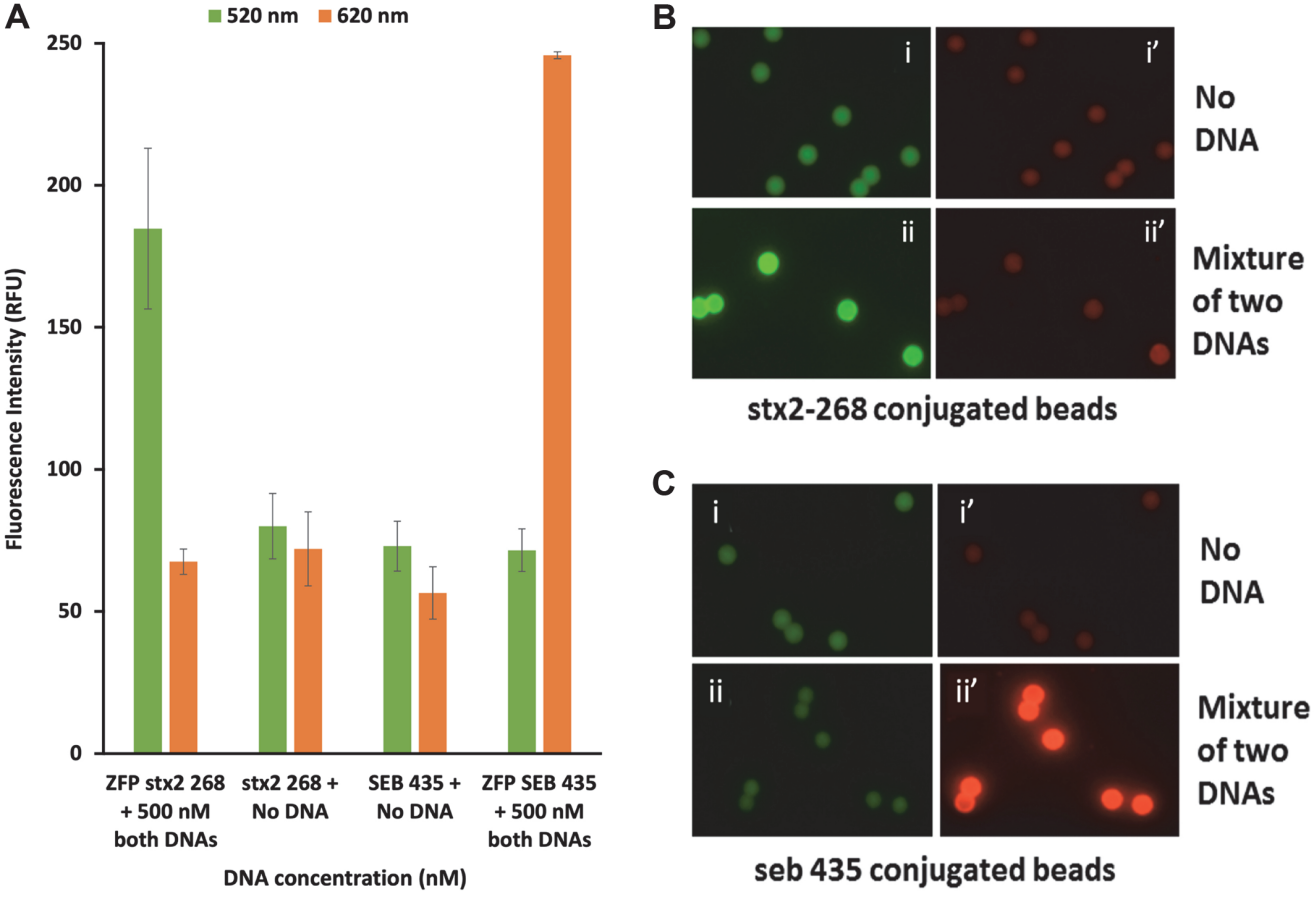
Specificity of engineered ZFPs (Stx2-268 and SEB-435). A mixture of two target DNAs was applied to both ZFPs. (**A**) The bar graph representing the fluorescence intensity of individual particles obtained from (**B, C**) images taken by fluorescence microscopy.

The DNA binding specificity was examined to investigate if engineered ZFPs are able to distinguish its own target DNA from non-target DNA. Mixtures containing 500 nM of both target DNAs were applied to each ZFP immobilized on the magnetic beads. As shown in Fig. 3, fluorescence signal was only observed when incubated with their respective ZFP because both ZFPs bound to only its cognate DNA in the presence of non-target DNA. Non-target DNA was washed away as shown in the image ii’ of Fig. 3B and the image ii of Fig. 3C since the signal intensity of non-target DNA is the same as the baseline autofluorescence of the beads. Thus, our system demonstrated high specificity.

### Multiplexed Detection

**Fig. 4:**
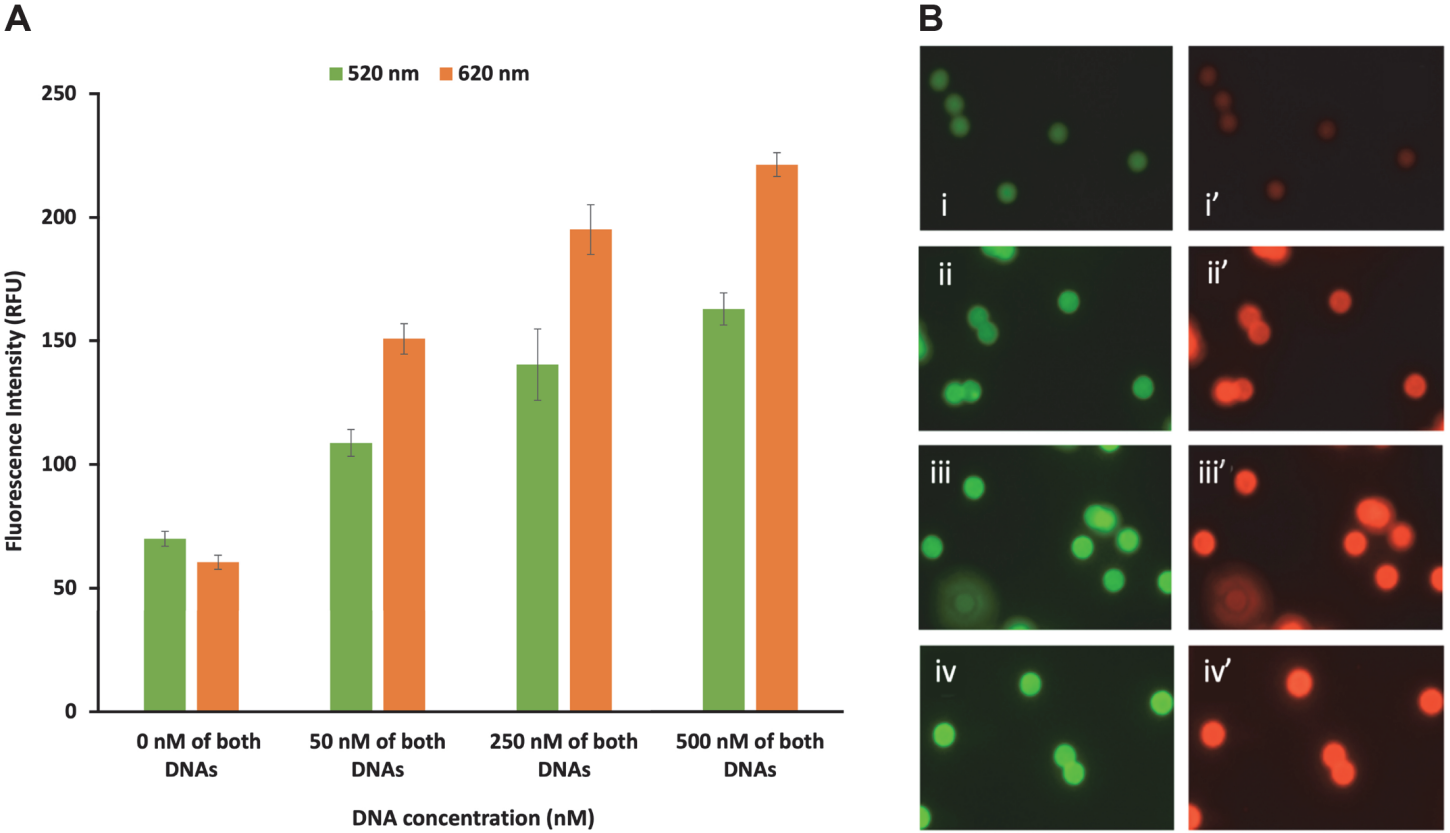
Multiplexed detection of target dsDNA by engineered ZFPs (Stx2-268 and SEB-435). (**A**) The bar graph representing the fluorescence intensity of individual particles obtained from (**B**) images taken by fluorescence microscopy (i and i’: 0 nM, ii and ii’: 50 nM, iii and iii’: 250 nM, iv and iv’: 500 nM of both DNA mixture applied to both ZFPs).

The capability of multiplexed detection of our system was investigated as shown in Fig. 4. Mixtures of both DNAs in various concentrations from 50 to 500 nM were applied to both ZFPs immobilized on magnetic beads. The fluorescence intensity of individual particles was obtained from the average of three independent experiments where data were collected ranging from 200 to 300 particles (1^st^ run), 150 to 200 particles (2^nd^ run), and 150 to 300 particles (3^rd^ run). The fluorescence signals from two different target DNAs exhibit different intensities simultaneously, indicating that each ZFP recognized its cognate DNA at the same time in the presence of two different target DNAs. The DNA-dose dependent signal was observed for both ZFPs. Our results demonstrated that the bead-conjugated ZFP-based method can detect multiple targets simultaneously, suggesting its potential for development into a simple and reliable method for multiplexed detection of pathogens.

## Conclusions

We have demonstrated a simple, rapid, and direct method for multiplexed detection of pathogens by isolating magnetic beads conjugated with ZFPs bound to pathogen-specific dsDNA. Our system allows for directly detecting pathogen-specific dsDNA, obviating the need for DNA denaturation and hybridization, and for efficiently isolating the protein-DNA bound complex through magnetic beads. The high specificity of our system has been demonstrated by the recognition of their own target DNA by ZFPs in the presence of non-target DNA. This bead- and ZFP-based detection system also demonstrated a multiplexing capability by detecting multiple target DNAs simultaneously. In addition, our bead-based assay provides clear visual detection after a 20 min incubation, which is faster than a 2-3 h process of PCR. Future studies will be carried out for further optimization to decrease the background noise and increase the ZFP concentration, leading to enhanced sensitivity. Since we have demonstrated in this study a proof-of concept for application of our system for detecting pathogenic DNA, cell lysates would be utilized for practical application of our future study. In our recently published report [33], engineered ZFPs were able to detect specific genes when using prepurified genomic DNAs of *E. coli* O157 and *S. aureus* as in real-world settings. Thus, we expect that our future study with cell lysates could potentially lead to promising results. In parallel, we will focus on integrating our system into a microfluidic chip for the purpose of developing POC application.

## Figures and Tables

**Table 1:** Sequences of the target DNA oligonucleotides.

Target	Sequence
Stx2-268	5’-GAC GGC TTG ATG TCT ATC AGG CGC GTT TTG ACC ATC TTC GGG TTTT CCC GAA GAT GGT CAA AAC GCG CCT GAT AGA CAT CAA GCC GTC-3’
SEB-435	5’-GAC GGT GTG ACC GAG CAT GAT GGA AAT CAA ATA GAT AAA CCC TTTT GGG TTT ACT TAT TTG ATT TCC ATC ATG CTC GGT CAC ACC GTC-3’

**Table 2:** Zinc finger recognition modules with their corresponding 3 bp DNA subsites, and the *k*_D_ values of zinc finger proteins.

ZFP	Position	Finger 6	Finger 5	Finger 4	Finger 3	Finger 2	Finger 1	*k*_D_ (nM)
Target site	268	GAA	GAT	GGT	CAA	AAC	GCG	1.98
Stx2-268		RSDDLVR	DSGNLRV	QSGNLTE	TSGHLVR	TSGNLVR	QSSNLVR	
Target site	435	GGA	AAT	CAA	ATA	GAT	AAA	0.3
SEB-435		QRANLRA	TSGNLVR	QKSSLIA	QSGNLTE	TTGNLTV	QRAHLER	
